# Glycosaminoglycans in osteoarthritis cartilage promote inflammation by preferential activation of the lipoxygenase rather than the cyclooxygenase pathway

**DOI:** 10.1186/s42358-025-00478-z

**Published:** 2025-09-26

**Authors:** Ana Carolina Matias Dinelly Pinto, Rodolfo de Melo Nunes, Vivian Louise Soares de Oliveira, Flavio Almeida Amaral, Francisco Airton Castro Rocha

**Affiliations:** 1 Postgraduate Program in Medical Sciences, Faculdade de Medicina da Universidade Federal do Ceará, Fortaleza, Ceará Brazil; 2 Centro Pasteur Fiocruz de Imunologia e Imuniterapia -Fiocruz CE, Fortaleza, Ceará Brazil; 3 Instituto de Ciências Biológicas da Universidade Federal de Minas Gerais, Postgraduate Program in Biochemistry and Immunology, Belo Horizonte, Minas Gerais Brazil; 4 Instituto de Biomedicina – Laboratório de Investigação em Osteoartropatias, Rua Coronel Nunes de Melo, 1315 -1º. Andar Rodolfo Teofilo – Fortaleza, Fortaleza, Ceará 60430-270 Brazil

**Keywords:** Glycosaminoglycans, Inflammation, Leukotrienes, Lipoxygenase, Osteoarthritis

## Abstract

**Aim:**

To determine whether glycosaminoglycans (GAG) of cartilage with osteoarthritis promote inflammation through activation of cyclooxygenase (COX)-2 and/or 5-lipoxygenase (ALOX5) pathways.

**Materials:**

GAG (50 µg/25µL) extracted from the cartilage of 30 patients subjected to total joint arthroplasty secondary to osteoarthritis (OA) or fracture or saline were injected intra-articularly (i.art.) into wild-type or ALOX5^−/−^ SV129 mice; cell counts as well as COX2 and ALOX5 gene expression were assessed in joint washes after 6 h.

**Results:**

GAG injection promoted acute cell migration, with predominance (> 85%) of mononuclear cells with GAG from women having greater activity than men. GAG injection significantly increased ALOX5 but not COX2 gene expression in cells of the joint washes, as compared to control. Injection of GAG from patients that sustained a fracture did not alter cell influx in ALOX5^-/-^ mice but cell counts were significantly lower following injection of GAG from OA patients (*P* = 0.0016).

**Conclusion:**

GAG promote inflammatory cell migration into joints through triggering of the 5-lipoxygenase pathway. Sex issues may be linked to an increased inflammatory potential of GAG from female OA patients.

## Background

Current knowledge suggests that activation of cells present in the synovium of patients with osteoarthritis (OA) promotes the release of inflammatory mediators that ultimately lead to joint damage [1]. Glycosaminoglycans (GAG) present in the extracellular cartilage matrix can behave as proinflammatory agents through inducing migration and activation of leukocytes, in addition to interacting with chemokines and cytokines [2]. Chondroitin sulfate (CS), which is the predominant GAG in the joint cartilage, may function as a damage-associated molecular pattern (DAMP) that couples to toll-like receptors (TLR) thereby activating innate immune mechanisms [3]. Modification of the structure and sulfation pattern of CS may alter the biological activity with an impact on the resulting inflammatory response [4]. We have recently shown that CS from patients subjected to arthroplasty secondary to OA display increased molar mass and decreased sulfation as compared to CS from patients that sustained a fracture, used as controls [5].

Prostaglandins and leukotrienes (LT) are formed following cleavage of phospholipids of the cell membrane and metabolism by the COX and LOX pathways. Whereas prostaglandins generated from COX2 activation are well-defined mediators in OA, the involvement of LT in OA is less well recognized [6]. The activation of the 5-lipoxygenase (ALOX5) enzyme is a rate-limiting step in LT formation by the conversion of the arachidonic acid into LTA_4_ which can lead to downstream production of LTB_4_ and the cysteinyl-LT, namely LTC_4_, LTD_4_, and LTE_4_. Activation of other enzymes, such as 15-lipoxygenase (ALOX15) leads to the release of other lipid mediators, including resolvins, which also display anti-inflammatory activity [7]. It was shown that LT are formed in OA joints [8] as well as increased LOX expression in dogs with hip OA [9]. On the other hand, deletion of the ALOX12/15 genes in mice accelerated the development of an experimental posttraumatic OA model [10]. These apparent discrepancies demand investigation in order to clarify the role, if any, of LT in the inflammatory process happening in joints affected by OA. Deciphering the relevance of LT to the pathophysiology of this disease is still an unmet need. Additionally, it could also foster studies to repurpose antagonists of cysteinyl-LT already in the market, with an acceptable safety profile, as alternatives to treat OA patients. Our present data show that injection of human GAG into naïve mouse joints promote influx of inflammatory cells which is higher after injection of GAG from OA patients as compared to GAG from healthy controls. This GAG-induced cell migration is linked to increased expression of the ALOX5 gene.

## Materials and methods

### Materials

Unless otherwise stated, all reagents were purchased from Sigma-Aldrich do Brasil S.A., São Paulo, Brazil.

### Collection of human cartilage samples

Samples from thirty (30) patients subjected to total joint arthroplasty in the Hospital Universitário Walter Cantídio (HUWC), Fortaleza - CE, Brazil, secondary to OA or fracture were collected. The clinical files and radiographies were evaluated by a senior rheumatologist (FACR) to confirm the OA diagnosis and exclude inflammatory arthropathy in patients with a fracture diagnosis [5]. GAG were extracted from human cartilage within less than 3 h post-surgery. GAG injected into the joints of the mice derived from a single patient. The protocol was approved by our local Ethics Committee (Protocol number 090.12.08). All patients signed a written informed consent prior to any procedure.

### GAG extraction and proteolysis

Cartilage samples were weighed after overnight drying (80 °C) and stored in acetone. Proteolysis was done by incubating 1 mg cartilage with 20 µL of a 0.4% w/v suspension of PROLAV 750^™^ (Prozyn, SP, Brazil) in Tris–HCl/NaCl 50 mmol/L / 150 mmol/L buffer (pH 8.0) [11]. The remaining proteins were precipitated with trichloroacetic acid to a final concentration of 10% w/v and centrifuged (10000 g/15 min at 25 °C). GAG were precipitated from the supernatant with two volumes of ethanol, followed by an overnight incubation at 4 °C and centrifugation (10,000 g/15 min at 15 °C), being dissolved in 20 µL of distilled water. Debris was weighted and the percentage of digestion was calculated as follows:

$$\begin{aligned}\mathrm{\%}\:\mathrm{d}\mathrm{i}\mathrm{g}\mathrm{e}\mathrm{s}\mathrm{t}\mathrm{i}\mathrm{o}\mathrm{n}\: =\:&[1\:-\:(\mathrm{w}\mathrm{e}\mathrm{i}\mathrm{g}\mathrm{h}\mathrm{t}\:\mathrm{o}\mathrm{f}\:\mathrm{d}\mathrm{e}\mathrm{b}\mathrm{r}\mathrm{i}\mathrm{s}\cr &/\mathrm{w}\mathrm{e}\mathrm{i}\mathrm{g}\mathrm{h}\mathrm{t}\:\mathrm{o}\mathrm{f}\:\mathrm{d}\mathrm{r}\mathrm{i}\mathrm{e}\mathrm{d}\:\mathrm{c}\mathrm{a}\mathrm{r}\mathrm{t}\mathrm{i}\mathrm{l}\mathrm{a}\mathrm{g}\mathrm{e}\left)\right]\:\times\:100\mathrm{\%}\end{aligned}$$

### GAG quantification

The GAG extract was separated on a 0.6% w/v agarose-gel electrophoresis in diaminopropane-acetate buffer (50 mmol/L; pH 9.0). The GAG were fixed in the gel through immersion in a 0.1% w/v cetyl-trimethylammoniun bromide solution for 2 h. The gel was dried and stained with 0.1% w/v toluidine-blue (in acetic acid: water: ethanol 1:49:50). C4S, C6S, and heparan sulphate standards were subjected to the same protocol. Quantification was made by densitometry (525 nm), providing µg CS/mg of dried cartilage.

### Animals

All animal procedures and experimental protocols were approved by the local ethics committee on animal experimentation at the Faculdade de Medicina of the Federal University of Ceará, Brazil (protocol number 113/07). All efforts were made to minimize animal suffering and the number of animals used. A total of 54 Swiss mice of either sex were housed in temperature-controlled rooms with 12-h light/dark cycles and free access to water and food. In addition, 12 SV129 mice with targeted disruption of the ALOX5 gene (ALOX^−/−^ mice) and 12 of their wild-type control background SV129 mice were used for analysis of ALOX5 experiments, as described below.

### Intra-articular injection

The average GAG content in the synovial fluid from OA joints has been estimated as 50 µg/mL [11]. Thus, naïve mice received, into the right knee joints, either a 25µL intra-articular (i.art.) injection containing 50 µg of GAG extracted from the cartilage of patients with an OA or fracture diagnosis or 25µL saline.

### Assessment of cell influx into joint aspirates

Animals were sacrificed under terminal anesthesia using i.m. ketamine/xylazine (50/10 mg/kg). The synovial cavity of the knee joints was washed 6 h following i.art. injections with 0.05 ml saline containing 10 mM EDTA and total cell counts determined using a Neubauer chamber. Differential cell counts were assessed using an Instant Prov™ staining kit (New ProvBrasil™). Migrated cells into the joints were collected after centrifugation (500 g/10min) and used to evaluate quantitative gene expression (see below).

### Analysis of cyclooxygenase-2 and 5-lipoxygenase expression

Cells present in joint exudates following injection of GAG or saline (control) were used for extraction of the mRNA which was reverse transcribed. The quantitative expression of COX2 and ALOX5 genes was evaluated using GAPDH gene as reference. The primer pairs sequences were:

#### Alox5

forward 5’- AGC TGC CTG CTG TGC ATC CC − 3’; reverse 5’ - CCC GGT GGC ATT GGC CTT GT – 3’.

#### Cox2

forward 5’ - AGA AGG AAA TGG CTG CAG AA – 3’; reverse 5’ - GCT CGG CTT CCA GTA TTG AG – 3’.

### 5-lipoxygenase knock-out experiment

Twenty-four SV129 mice were bred and maintained at the Instituto de Ciências Biológicas of the Universidade Federal de Minas Gerais. Briefly, either twelve (12) wild-type SV129 mice or twelve (12) SV129 animals subjected to targeted disruption of the 5-lipoxygenase gene, namely ALOX5^-/-^ SV129 mice, received intra-articular injection of GAGs (50 µg/25µL) isolated from either OA or fracture patients, or the same volume of saline (control) into their right knee joints. After 6 h, animals were sacrificed under terminal anesthesia (ketamine/xylazine 50/10 mg/kg i.m.) and the knee joints were washed for assessment of cell counts.

### Statistics analysis

Results are presented as means ± SEM; for multiple comparisons between means we used one-way ANOVA followed by Tukey’s test, respectively; *P* < 0.05 was considered as significant.

## Results

Patient demographics and cartilage samples were recently reported. Briefly, 30 samples were collected, comprising 20 female (66.6%) and 10 (33.3%) male patients (Table 1). Age was equally distributed among fracture and OA groups. The amount of GAG extracted varied with the quantity of cartilage collected, regardless of patient’s sex [5].

**Table 1:** Demographics and clinical data of osteoarthritis (OA) or fracture (control) samples

	Groups	Fracture	OA
Age (Mean ± SD)		67 ± 5	67 ± 4
Gender	Female	10	10
	Male	6	4
Joint	Hip	10	10
	Shoulder	6	4

### Analysis of cell influx into joint lavage

**Fig. 1:**
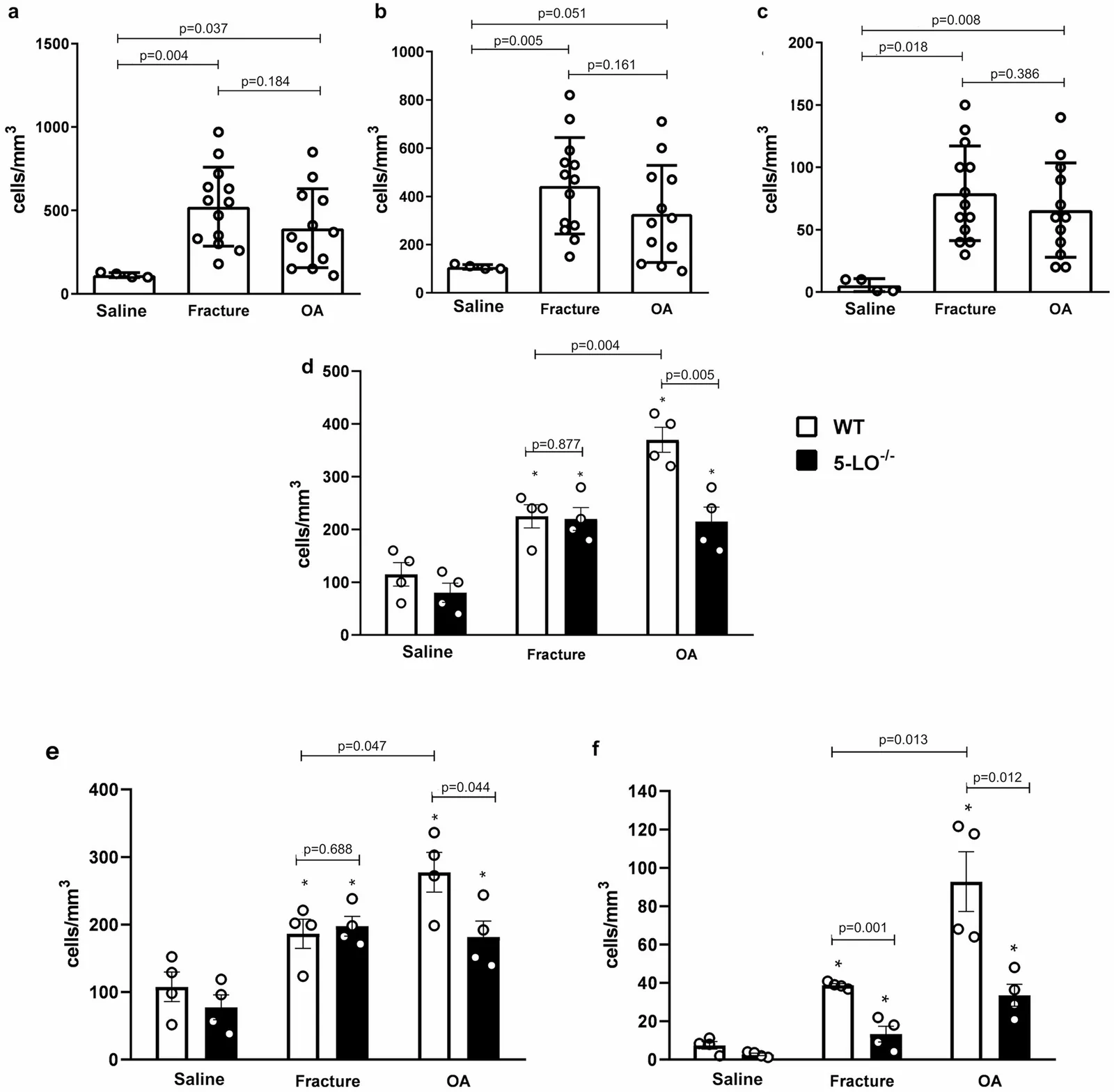
Glycosaminoglycan (GAG)-induced cell migration into naïve joints. GAG solutions (50 µg/25µL) extracted from the cartilage of patients subjected to arthroplasty due to osteoarthritis (OA) (*n* = 12) or fracture (*n* = 13) were injected into wild type Swiss mouse joints (**a**-**c**) or SV129 [d-f; WT, wild-type or ALOX5^−/−^ (Genetically modified SV129 mice lacking ALOX5 gene)]. Cell counts were evaluated in joint exudates collected 6 h post injection. Data represent mean ± SEM of total (**a**, **d**), mononuclear (**b**, **e**), and polymorphonuclear cell counts (**c**, **f**) of *n* > 4 animals/group; 4 SV129 wild type or ALOX5^−/−^ animals received GAG from OA (*n* = 2) or fracture (*n* = 2) patients; one-way ANOVA followed by Tukey’s test; p values are indicated whereas *means *p* < 0.05 as compared to saline

Total cell count following injection of GAG was significantly higher with female samples: 784.4 ± 386.5 and 408.2 ± 179.2 cells/mm^3^ for female (*n* = 10) and male (*n* = 6) fracture patients (*p* = 0.045; 95% CI -63.4–743.8) with 666.8 ± 370.1 and 269.4 ± 193.5 cells/mm^3^ for female (*n* = 10) and male (*n* = 4) OA patients (*p* = 0.035; 95% CI -40.89–835.8), respectively. Figure 1a-c illustrate an increase in cell counts in wild-type Swiss mice that received GAG from OA (*p* = 0.037; 95% CI 93.46–727.2) or fracture (*p* = 0.004; 95% CI -39.38–600.5) patients, as compared to animals subjected to i.art. saline injection, with predominance (> 85%) of mononuclear cells. Cell counts were similar when comparing animals that received GAG from OA or fracture patients (*p* = 0.184; 95% CI -92.11 to 351.6) as seen in Fig. 1a, b and c show that data for both polymorphonuclear and mononuclear cell counts reproduced the results observed with total cell counts. Figure 1d-f show that injection of GAG from fracture or OA patients into the knee of wild-type or ALOX5^−/−^ mice induced a significantly higher cell influx, as compared to values in naïve joints that received saline. Interestingly, in the wild-type mice of the SV129 strain, injection of GAG from OA patients induced a significantly higher cell influx, as compared to joints that received GAG from fracture patients (*p* = 0.004; 95% CI -22.46 - -6.53; 1d). However, in the ALOX5^−/−^ SV129 group of mice, cell influx was similar after injection of GAG from OA or fracture patients (*p* = 0.877; 95% CI -7.07–8.07; 1d). The total number of cells appear lower in SV129 mice, as compared to Swiss mice. Notably, cell counts in the joints of ALOX5^−/−^ mice that received GAG from OA patients was significantly lower as compared to cell counts in wild type SV129 mice (*p* = 0.005; 95% CI 6.59–24.41; 1d). The number of mononuclear cell counts in joint washes reproduced the data with total cell counts. Thus, mononuclear cell counts were significantly higher in animals that received GAG from OA patients as compared to those that received from fracture patients (*p* = 0.047; 95% CI -18.01 to -0.183; 1e), mononuclear cell counts were similar after injection of GAG from fracture patients in wild type SV129 or ALOX5^−/−^ mice (*p* = 0.688; 95% / IC -7.48–5.28; 1e) and those cell counts were significantly lower after injection of GAG from OA patients in ALOX5^−/−^ mice, as compared to wild type SV129 mice (*p* = 0.044; 95% CI 0.35–18.78; 1e). Polymorphonuclear cell counts were also significantly higher after GAG injection into the joints, as compared to saline, regardless of analyzing wild type SV129 or ALOX5^−/−^ mice. Injection of GAG from OA patients induced higher cell influx as compared to GAG from fracture patients in wild-type SV129 mice (*p* = 0.013; 95% CI -9.20 - -1.60; 1f). Polymorphonuclear cell counts were higher in wild-type SV129 as compared to ALOX5^−/−^mice, whether injecting GAG from fracture or OA patients (*p* = 0.001 95% CI 1.37–3.10 and *p* = 0.012; 95% CI 1.87–9.98, respectively; 1f). We should also mention that no animals died during the experiments, nor was any change in behavior observed.

### Expression of COX2 and ALOX5 genes

**Fig. 2:**
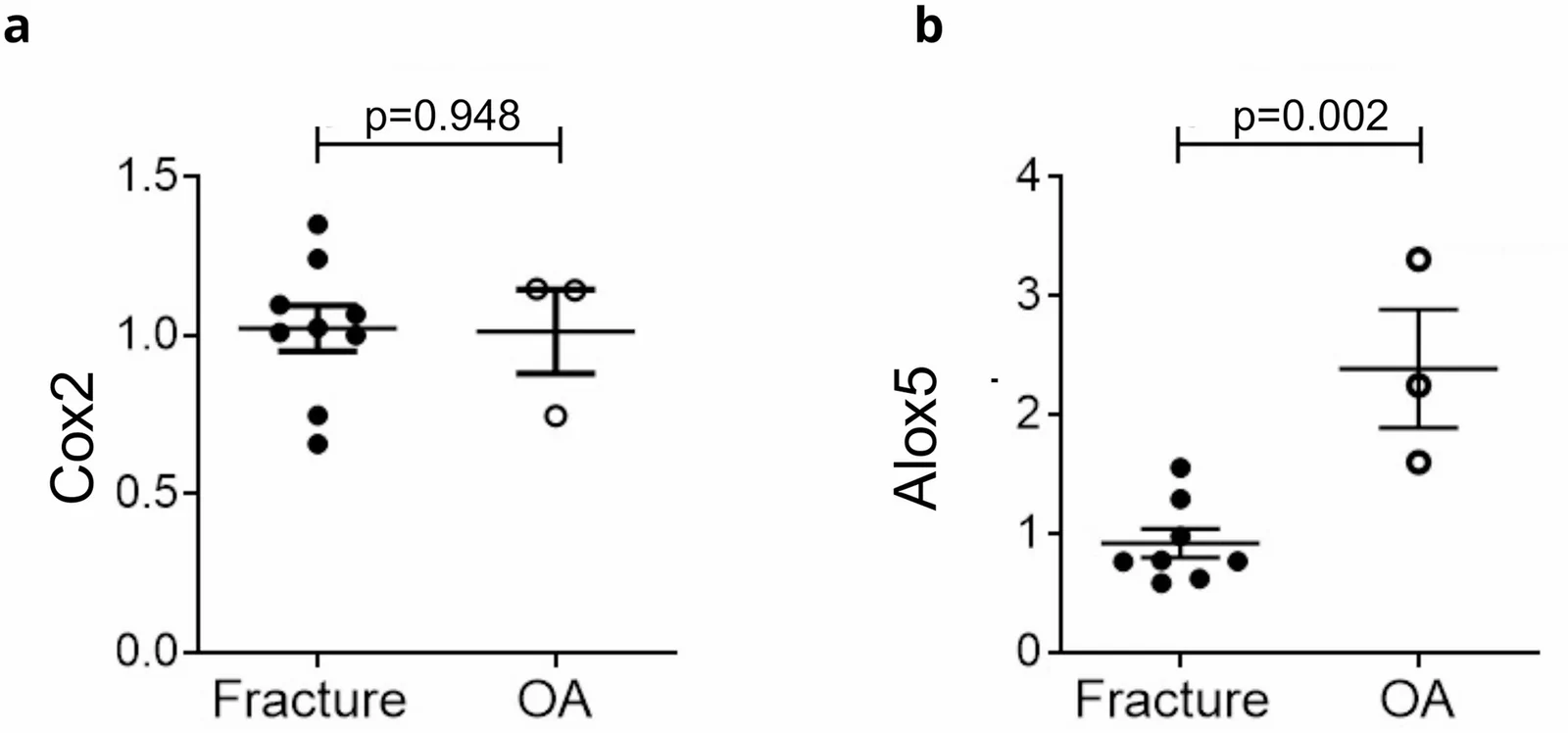
Relative expression of cyclooxygenase-2 (COX2) and 5-lipoxygenase (ALOX5) genes in mouse joint lavage cells following GAG injection. GAG solutions (50 µg/25µL) extracted from the cartilage of patients subjected to arthroplasty due to osteoarthritis (OA) (*n* = 3) or fracture (*n* = 9) were injected into naïve mouse joints and mRNA of cells collected from joint lavages after 6 h were assessed for expression of the COX2 and ALOX5 genes using GAPDH as a reference gene, using RT-qPCR analysis. Data represent mean ± SD of relative gene expression in OA and fracture groups evaluated using Student’s “t” test

Figure 2a illustrates that the relative expression of the COX2 gene in cells collected from joint exudates was similar in both fracture and OA groups as compared to the reference gene (*p* = 0.948; 95% CI -0.3149–0.3344). Figure 2b illustrates a statistically significant increase (*p* = 0.002; 95% CI -2.242 - -0.6941) in the expression of the ALOX5 gene in cells from the joint lavage of animals that received GAGs from patients with OA, as compared to samples from patients of the fracture group.

## Discussion

Our data demonstrate that GAG obtained from patients subjected to arthroplasty promote cell migration after injection into mouse joints. GAG injection was associated with an increase in the expression of the ALOX5 gene in cells collected from the joint washes, though not altering the expression of the COX2 gene. To the best of our knowledge, this is the first demonstration that GAG from human joints, namely CS, induce inflammation that involves activation of the ALOX5 pathway.

Inflammatory cells and chondrocytes were shown to display GAG receptors and GAG are also able to increase the expression of specific cytokine receptors [4]. Pathophysiological events triggered by CS were associated with the development of tissue lesions in the central nervous system of patients affected by multiple sclerosis [12]. The fact that we injected GAG in a concentration equivalent to levels found within normal joints points to a possible clinically meaningful mechanism [11]. We may speculate that cartilage damage in a knee joint occurring secondary to weight-bearing activities is a very likely continuous traumatic damage mechanism happening in daily life activities. Persistent release of GAG might activate synoviocytes thus promoting chronic low grade inflammation. Interestingly, cell influx in joints that received GAG from female patients was higher, regardless of being from OA or non-OA individuals. Qualitative changes have been shown in GAG from patients suffering from arthritic diseases. In this regard, our group has recently shown that GAG from OA patients display increase in molar mass and reduced sulfation, a change that was associated with a reduction in the modulus of the Zeta potential, an essential property of GAG. We also observed that the concentration of GAG was higher in the articular cartilage from females who also had a trend towards lower sulfate content, as compared to males, suggesting sex-linked phenotype differences [5]. It was also reported that production of ALOX5 metabolites was higher in cells from females, and that the pharmacological effect of inhibitors of both ALOX5 activating protein (FLAP) and ALOX5 varied by sex, being more effective in females. Notably, diseases where LT play a prominent role, including asthma and allergic rhinitis, are more prevalent in females and LT may also account for sex differences in multiple sclerosis patients [13]. Although speculative, since women are more commonly affected by OA, our results indicate that an enhanced though yet unexplored higher inflammatory potential of GAG from female patients via the LOX pathway may be linked to the increased OA prevalence in this sex.

We also found that injection of GAG from OA patients induced an increased cell influx into the joints of SV129 wild-type mice, as compared to GAG from fracture patients. However, this increased cell influx was reversed when GAG from OA patients were injected into ALOX5^−/−^ mice, affecting both mononuclear and polymorphonuclear cell migration. Coupled to the increased expression of the ALOX5 gene in cells migrated following injection of GAG into the joints mentioned above, these data suggest that the inflammatory role of GAG, particularly in OA joints, is likely to be associated with activation of the ALOX5 pathway. The conversion of arachidonic acid to LTA_4_ leads to the formation of potent proinflammatory LT, such as LTB_4,_ as well as the cysteinyl-LT. On the other hand, lipoxin A_4_ may also be formed, eventually leading to the formation of pro-resolving compounds, which display anti-inflammatory activity. Also, activation of 12/15 LOX results in the formation of anti-inflammatory compounds and deletion of the genes encoding these enzymes worsened joint damage in mice subjected to experimental OA, thus illustrating the complexity of the biological activity of LOX-derived compounds in inflammatory disorders [7]. Although we did not determine which LOX compounds were generated, our results suggest that the in vivo exposure of joint tissues to CS triggers inflammation, as evidenced by acute cell influx into the joints. Interestingly, GAG injection, both from OA or fracture patients, did not modify the expression of the COX2 gene in cells collected from the joint washes. Time-sampling could explain this result given that we only measured gene expression in cells collected 6 h after GAG injection. We also did not evaluate gene expression in cells within the synovial tissue, neither in chondrocytes nor in subchondral bone cells. While nonsteroidal anti-inflammatory drugs (NSAID) are the most classic painkillers used in OA, COX2 blockade is not linked to inhibition of joint damage. LT are major players in inflammation, with LTB_4_ being a potent chemoattractant that also activates other inflammatory cells [6, 7]. COX inhibition by NSAID, on the other hand, was claimed to increase the release of LT via a deviation of substrate availability [14]. Although speculative, one may thus argue that prolonged inhibition of COX2 by NSAID use in OA patients might in fact favor LT production thereby promoting joint damage. Although there are no specific anti-arthritic drugs that target LT in clinical practice, a component of *Boswellia serrata*, namely 3-*O*-acethyl-11-keto-β-boswellic acid, was shown to be superior to placebo in providing pain relief in a phase II trial in OA patients. That effect was linked to inhibition of ALOX5 [15].

Our study has limitations, including the relatively low number of GAG samples. Ethical issues are a limitation in sample collection, particularly when working with human material. Thus, our data can be regarded as illustrative of a pathophysiological mechanism that awaits further confirmation in a clinical environment. Also, we did not evaluate data with chronically, persistently inflamed joints, which could mimic daily life damage, due to limitations regarding sample (GAG) availability. Repeated GAG injections with different time sampling as well as analysis of gene expression at the tissue level or assaying synovial fluid level of inflammatory mediators in longer time points could help determine whether this strategy reproduces a sustained inflammatory response contributing to joint damage. In conclusion, this is the first in vivo demonstration that GAGs induce cell influx into joints, which is more pronounced in women, being associated with increased expression of the ALOX5 gene. The linkage of GAG-induced cell influx to sex issues regarding ALOX5 activation deserves further studies. The existence of safe compounds that interfere with the LOX pathway currently marketed for other purposes, such as sodium montelukast [16], opens the possibility of repurposing strategies for designing clinical protocols to treat OA with such compounds.

## Data Availability

No datasets were generated or analysed during the current study.

## Abbreviations

ALOX5^−/−^: Genetically modified mice lacking ALOX5 gene
CS: Chondroitin sulfate
COX: Cyclooxygenase
DAMP: Damage-associated molecular pattern
GAG: Glycosaminoglycans
i.art: Intra-articular
NSAID: Nonsteroidal anti-inflammatory drugs
OA: Osteoarthritis
ALOX5: 5-lipoxygenase
